# The Remediation of Arsenic-Contaminated Soil by *Pteris vittata* L. Facilitates the Recovery of Soil Bacterial Diversity and Network Complexity

**DOI:** 10.3390/microorganisms13102316

**Published:** 2025-10-07

**Authors:** Feng Li, Jinhua Liu, Tao Tian, Bin Deng, Haifeng Xiao

**Affiliations:** 1 School of Chemistry and Environmental Science, Xiangnan University, Chenzhou 423000, China; lifeng214@xnu.edu.cn (F.L.); liujinhua@xnu.edu.cn (J.L.); tiantaoxnxy@163.com (T.T.); 2 Hunan Provincial Key Laboratory of Xiangnan Rare-Precious Metal Compounds and Applications, Chenzhou 423000, China

**Keywords:** arsenic contamination soil, *Pteris vittata* L., phytoremediation, bacterial diversity and communities, molecular ecological networks

## Abstract

The remediation of contaminated soils is essential for restoring land productivity and soil health. *Pteris vittata* L., an arsenic hyperaccumulator, has been widely used for phytoremediation, yet its ecological effects on soil systems remain insufficiently understood. In this field study, we evaluated the influence of *Pteris vittata* L. remediation on soil physico-chemical properties, microbial diversity, and molecular ecological networks. The results showed that long-term arsenic contamination significantly reduced soil total carbon, total nitrogen, and available phosphorus, simplified bacterial network structures, and markedly altered the keystone taxa that maintain microbial interactions. In contrast, soils under *Pteris vittata* L. remediation exhibited higher nutrient availability, greater bacterial diversity, and more complex microbial networks than contaminated soils, indicating partial recovery of ecosystem functions. These findings demonstrate that *Pteris vittata* L. remediation can mitigate arsenic-induced soil degradation and provide an important scientific basis for assessing the long-term impacts of arsenic contamination and the role of remediation measures in soil health evolution.

## 1. Introduction

Arsenic pollution has emerged as a global environmental issue of considerable concern, as it significantly impacts agricultural sustainability, soil ecosystem health, and human health, which cannot be overlooked [1,2,3]. The average concentration of total arsenic in contaminated soils worldwide ranges from 126 to 1600 mg kg^−1^ [4]. However, the degree of contamination varies significantly among different soil types; for instance, sandy soils and clayey soils exhibit distinct behaviors regarding arsenic retention and migration. Compared to coarse-textured soils, fine-textured soils generally retain more arsenic due to their higher mineral and organic matter content [5]. Arsenic can inhibit plant growth and development by destroying chlorophyll in plant cells and affecting the leaf net photosynthetic rate, leading to decreased crop yield and quality, and even plant death [6]. Arsenic contaminants can also accumulate in food crops and plants, subsequently entering the human body through the food chain [7,8], thereby directly endangering human health and potentially causing cancer [9,10,11]. Elevated concentrations of arsenic pollution in the soil environment can also alter microbial communities, reduce biomass and activity, and consequently affect the functions of the entire soil ecosystem [12,13]. Therefore, effective remediation of arsenic-contaminated soils is essential for maintaining sustainable soil production functions and ecosystem services.

*Pteris vittata* L., recognized as an excellent hyperaccumulator, can efficiently absorb and accumulate arsenic from the soil and has been widely applied in the remediation of arsenic-contaminated soils, demonstrating significant effectiveness in reducing arsenic concentrations [14,15,16,17,18,19]. *Pteris vittata* L. can accumulate arsenic in its fronds at remarkably high levels, with studies reporting concentrations exceeding > 22,000 mg arsenic kg^−1^ dry weight [19]. It has also been reported that *Pteris vittata* L. can uptake over 60% of available arsenate from the soil within a growing season [19], demonstrating its efficacy in phytoremediation. However, most previous studies have focused only on the removal rate of arsenic pollutants. The ecological restoration effects and the underlying mechanisms—such as how *Pteris vittata* L. improves soil quality by altering rhizosphere microbial communities—remain insufficiently investigated. Studies have shown that as *Pteris vittata* L. absorbs arsenic, it influences the abundance and diversity of its rhizosphere microbial populations, leading to the enrichment of specific functional groups that can further assist in detoxifying soil contaminants, thereby improving soil quality [18,20]. To our limited knowledge, few studies have comprehensively evaluated the continuous recovery differences in soil ecological systems between arsenic-contaminated soils and *Pteris vittata* L. remediated soils from the perspectives of both soil nutrients and soil organisms under field conditions. It is important to note that the utilization of *Pteris vittata* L. for phytoremediation has limitations. Factors such as soil texture and clay content can significantly restrict arsenic uptake by *Pteris vittata* L. [21], while climatic conditions may also influence its growth and efficiency [22]. These limitations underscore the need for further research to identify optimal conditions for effective phytoremediation and to explore complementary remediation strategies.

Soil organisms, particularly soil microorganisms, have long been recognized as effective biological indicators for assessing soil restoration progress and reflecting the overall status of soil health [23,24]. Continuous monitoring of changes in soil microbial diversity and communities can provide critical insights for revealing the regulatory effects of plant restoration on soil ecosystems. Soil microorganisms are the core of regulating soil ecosystem functions. They participate in the absorption and transport of arsenic via phosphate channel proteins [25]. Excessive arsenic absorption by microorganisms under prolonged exposure to high concentrations of arsenic pollution will inevitably affect their communities. Studies have demonstrated that arsenic stress significantly affects the communities of Acidobacteria and Nitrospirae [12]. While high concentrations of arsenic inhibit the growth of sensitive strains, the growth performance of certain arsenic-tolerant strains often improves under stress conditions. For instance, the Rhodococcus strain BCP1 exhibits enhanced growth when exposed to sublethal concentrations of As(V) [26]. Bacteria such as Klebsiella pneumoniae and Enterobacter can grow even at high arsenic concentrations (300 mg L^−1^ or higher), indicating that they possess robust resistance mechanisms [27]. These characteristics of microorganisms will reshape the entire community structure in response to arsenic pollution. However, few studies have previously considered the effects of arsenic pollution on the interactions between soil microbial species [28]. Soil microbial species do not exist in isolation. They interact through competition or cooperation to form complex communities. These inter-species interactions may be critical for maintaining community structure and soil ecosystem functions [29]. Therefore, it is imperative to further investigate the impacts of arsenic contamination and remediation on the interactions between soil microbial species.

In recent years, molecular ecological networks (a systems biology approach based on high-throughput sequencing data, used to analyze and visualize the interrelationships among different species (or taxonomic units) within microbial communities) of soil microorganisms have emerged as a powerful tool for deciphering microbial community interactions and are frequently employed to reveal the effects of environmental stresses, such as heavy metal pollution and land use on associations [29,30,31]. Alterations in the topological structure of molecular ecological networks influence ecosystem functions and stability [32,33], with greater network complexity generally associated with enhanced ecosystem stability [33,34]. Therefore, employing microbial molecular ecological networks to assess the sustained effects of long-term arsenic contamination and *Pteris vittata* L. remediation is significant for elucidating soil quality development. Additionally, under field conditions, arsenic pollution stress often persists for decades or even centuries [8,35]. However, few studies have continuously monitored changes in soil microbial diversity, community composition, and network structure in response to arsenic pollution over several years. Consequently, exploring the responses of microorganisms to arsenic contamination and remediation over consecutive years is also of considerable reference value for assessing the long-term changes in soil recovery and health.

This study aims to investigate the evolution of soil quality following remediation of long-term arsenic-contaminated soil by comparing differences in nutrient content, microbial diversity, community composition, and molecular ecological network complexity between arsenic-contaminated soil and *Pteris vittata* L. remediated soil. By doing so, we aim to provide a comprehensive understanding of the ecological risks associated with arsenic contamination and offer valuable insights into the soil ecological processes affected by arsenic pollution. This research will contribute to the understanding of soil health quality evolution and sustainable development, with the findings expected to have significant scientific implications for assessing the effects of long-term arsenic contamination and remediation.

## 2. Materials and Methods

### 2.1. Overview of the Study Area

The study site is located in Dengjia Tang Town, Chenzhou City, Hunan Province, China (25°48′ N, 113°02′ E) (Supplementary Figure S1). The region is characterized by a subtropical low hot valley climate, with an average annual temperature of 19 °C and an average annual precipitation of 1696 mm. The coldest month is January, with a mean temperature of 6.4 °C, while the warmest month is August, with a mean temperature of 28.3 °C. The climate exhibits distinct dry and rainy seasons, with precipitation primarily concentrated in spring and summer. The elevation of the study site is about 185 m. According to the World Reference Base for Soil Resources (WRB) classification, the soil type in this study area is Cambisols [36]. In the Chinese soil classification system, it belongs to the Red Soil, Ferralosols order. Soil texture is Loamy clay. The criteria for determining soil texture and information on soil particles for different treatments are presented in Supplementary Tables S1 and S2. In 1999, an arsenic product company dumped the waste residue produced from production in the natural depressions near the factory, resulting in varying degrees of arsenic contamination in the surrounding water, soil and crops, and hundreds of nearby residents suffered from arsenic poisoning, leading to the abandonment of approximately 50 hm^2^ of rice fields and vegetable fields in the area. In 2001, Professor Chen Tongbin from the Institute of Geographic Sciences and Natural Resources Research, Chinese Academy of Sciences, led a team to carry out *Pteris vittata* L. remediation experiments in the area, establishing the world’s first arsenic-contaminated soil phytoremediation demonstration base (1 hm^2^). In April 2002, after plowing the arsenic-contaminated soil, they planted *Pteris vittata* L., which was planted at a spacing of 40 cm × 40 cm, and mowed once a year in December, leaving a stubble height of 7.5 cm after mowing, and conducted continuous soil remediation for 6 years (from 2002–2008), greatly reducing the total arsenic content in the arsenic-contaminated soil [37]. Before soil remediation, the soil types and land use patterns were basically consistent across the three treatments. Currently, all three sample plots are abandoned natural grasslands, with herbaceous plants as the main vegetation. Detailed information on the vegetation can be found in Supplementary Table S3. Unfortunately, after the project ended, large areas of the region remained unremediated. There has also been a lack of continued monitoring on how the recovery status of soils differs between the unremediated areas and the remediated areas.

### 2.2. Soil Sampling

The sampling area in this study was divided into three regions, namely the arsenic-contaminated region, the *Pteris vittata* L. remediated region, and the control region. The contaminated region and remediated region were located downstream of the arsenic product factory, while the control region was located upstream of the factory. The distances of the three sampling areas from the contamination source were 1.5 km, 1.0 km, and 1.3 km, respectively. Since the arsenic contamination incident, the land use status of the study area has remained abandoned. Soil sampling was carried out annually in October from 2021 to 2023. During the sampling period, the mean temperature was approximately 20 °C, and no heavy rainfall occurred in the week preceding sampling, thereby preventing excessive soil moisture. Ring knives were used for sampling the surface soil at a depth of 0–15 cm. To maintain the independence of the samples, the distance between each sampling point was at least 20 m, with 10 repetitions in each region and 30 soil samples each year and 90 samples in total. The soil samples were placed in ice boxes, brought back to the laboratory quickly, gently mixed, and sieved through a 2 mm mesh to remove roots and pebbles. The soil was then divided into two parts, one part for the measurement of total and bioavailable arsenic and physicochemical properties, and the other part for soil bacterial community analysis.

### 2.3. Determination of Total Arsenic and Bioavailable Arsenic in Soil

The total arsenic content in soil was determined using the Chinese national standard GB/T22105 method [38]. The specific process is simply described as follows: The soil samples were naturally air-dried indoors, ground using an agate mortar, and sieved through a 100-mesh sieve. The sieved soil was digested using a digestant (1 part nitric acid (ρ = 1.42 g mL^−1^) mixed evenly with 3 parts hydrochloric acid (ρ = 1.42 g mL^−1^) (the procedure for microwave digestion is as follows: first, increase the temperature from room temperature to 120 °C over 7 min and maintain it at 120 °C for 3 min. Then, increase the temperature from 120 °C to 180 °C over 10 min and maintain it at 180 °C for 15 min), and then diluted 1:1 with water (GB/T22105), and the digested solution was determined for arsenic concentration using atomic fluorescence spectrometry.

The extraction of bioavailable arsenic was conducted using the AB-DTPA method [39]. The procedure involved accurately weighing 8 g of soil sample and adding 50 mL of AB-DTPA extraction solution (prepared by dissolving 1.967 g of DTPA (diethylenetriaminepentaacetic acid) in 800 mL of water containing 2 mL of ammonia solution (NH_3_·H_2_O), followed by the addition of 79.06 g of NH_4_HCO_3_. After most of the DTPA had dissolved, the pH was adjusted to 7.3 ± 0.2 using concentrated HCl, and the solution was transferred to a 1000 mL volumetric flask and made up to volume). The mixture was shaken at a frequency of 150 r·min^−1^ at 25 ± 2 °C for 2 h, and the extract was filtered through a 0.45 μm filter membrane. 10 mL of the filtrate was taken, and 1 mL of concentrated HNO_3_ was slowly added with continuous shaking until no more bubbles formed. The arsenic concentration in the solution was determined using atomic fluorescence analysis.

Quality control and assurance: To ensure accuracy and precision, analytical blanks and duplicate samples were included in each batch of analysis. A certified reference material (GBW07405, stream sediment, National Research Center for Certified Reference Materials, China) was analyzed alongside soil samples [40], and the recovery of arsenic ranged between 95% and 105%. Instrument calibration was performed using multi-point standard curves prepared from arsenic standard solutions (National Standard Material Center, China). The method detection limit (MDL) and limit of quantification (LOQ) for arsenic were 0.1 μg L^−1^ and 0.3 μg L^−1^, respectively. All analyses were conducted in triplicate, and the relative standard deviation (RSD) was below 5%.

### 2.4. Soil Nutrient Determination

Soil total C and N were measured using a Vario MAX CN (Elementar, Frankfurt, Germany) with 1 g of air-dried soil passed through a 0.15 mm diameter mesh [41]. Soil available K was extracted with 1 mol L^−1^ CH_3_COONH_4_ (soil:solution 1:10, 30 min shaking) and measured using an iCAP6300 (Thermo Fisher Scientific, Waltham, MA, USA) [41]. Soil available P (AP) was extracted with 0.025 mol L^−1^ HCl and 0.03 mol L^−1^ NH_4_F (soil:solution 1:10, 30 min shaking) and measured by continuous flow autoanalysis [41]. Soil pH was determined by sampling pH meter (Mettler-Toledo FE20 e FiveEasy Plus™, Switzerland). The methodology is as follows: Weigh 10.0 g of soil sample into a 50 mL beaker, add 25 mL of ultrapure water, seal the beaker with sealing film, and stir vigorously with a magnetic stirrer for 2 min. Let it stand for 30 min, and then measure the pH within the next hour [42].

### 2.5. DNA Extraction and PCR Amplification

Total soil DNA was extracted using the DNeasy^®^ PowerSoil^®^ Kit (QIAGEN, Hilden, Germany) following the manufacturer’s protocol. For PCR amplification, the bacterial V4-V6 primer pair (Forward primer, 515F:5’-GTGYCAGCMGCCGCGGTAA-3’; Reverse primer, 926R: 5’-CCGYCAATTYMTTTRAGTTT-3’ [43]) was used, which synthesized an eight-base barcode at the 5′ end of the forward primer. PCR program including an initial 4-min denaturing step at 95 °C; 35 cycles of denaturation at 95 °C for 30 s, annealing at 56 °C for 30 s, and extension at 72 °C for 45 s; and a final extension step at 72 °C for 5 min. The target size of the amplified PCR product was approximately 411 bp. The 50-μL reaction mixture consisted of 1× PCR buffer (TaKaRa, Shiga, Japan); 50 ng of DNA template, quantified using Qubit^®^ 2.0 (Life Technologies, Carlsbad, CA, USA); 10 pmol L^−1^ forward and reverse primers; 200 μmol L^−1^ dNTP mix; and 2.5 U Ex Taq DNA polymerase (TaKaRa). Purified PCR products were sent to Novogene Biotechnology Co., Ltd. (Tianjing, China) for sequencing with the Illumina MiSeq platform (read length 2 × 250 bp).

### 2.6. Determination of Operational Taxonomic Units

The raw MiSeq reads were merged using the fastq_mergepairs command in USEARCH 64-bit [44] and subsequently quality-filtered. Sequence quality filtering involved removing barcodes and deleting certain reads (sequence quality below Q30, fragment lengths less than 100 bp, and chimeras). The filtered reads were dereplicated with the fastx_uniques command, and the unoise3 command was used to cluster them into Zero-radius Operational Taxonomic Units (ZOTUs) (For ease of understanding, the term OTU will still be used elsewhere in the article) [45]. In addition, low-abundance OTUs (OTU abundance lower than 0.1%) were removed from the OTU table using the min_otu_freq command. For analysis of bacterial sequence data, the SILVA 119 SSU Parc database (www.arb-silva.de, accessed on 1 May 2024) was used for BLAST (Version 2.14.1+).

### 2.7. Construction of Molecular Ecological Networks

Using the above-constructed OTU table, molecular ecological networks were constructed for each treatment in different years based on random matrix theory (RMT). Network construction and acquisition of network property parameters were completed on the Molecular Ecological Network Analyses Pipeline (MENA) website (http://ieg4.rccc.ou.edu/mena, accessed on 1 May 2024) [46]. The topological properties of the network were characterized by indices such as number of nodes, links between nodes, connectivity, average geodesic distance, average clustering coefficient, and modularity. Network construction mainly included four main steps: data collection, data transformation/standardization, calculation of pairwise similarity matrix, and adjacency matrix based on RMT method. First, the collected ZOTU relative abundance data were log-transformed and uploaded in the required format. Then, the Pearson correlation coefficient between two transformed species was calculated to construct a correlation matrix, the absolute value of which was taken to convert the correlation matrix into a similarity matrix. Finally, the default similarity threshold was applied to obtain an adjacency matrix encoding the connection strength between each pair of nodes. The network files and corresponding node data files were imported into Gephi software (Version 0.9.2) for network visualization. Nodes were divided into 4 major types using the indices of among-module connectivity (Pi) and within-modules connectivity (Zi): network hubs (Pi > 0.62 and Zi > 2.5), module hubs (Pi ≤ 0.62 and Zi > 2.5), connectors (Pi > 0.62 and Zi ≤ 2.5), and peripherals (Pi ≤ 0.62 and Zi ≤ 2.5). Finally, using the Maslov-Sneppen method [47], 100 random networks were reconstructed by reconnecting nodes in different positions of the original network without changing the number of original nodes and links.

### 2.8. Statistical Analysis

To investigate the effects of arsenic contamination and time on arsenic concentrations, soil physicochemical properties, and bacterial diversity in soil, the data were log-transformed or square root-transformed as needed to achieve normality and eliminate heteroscedasticity. Two-way ANOVA was used to detect the significant effects of time and arsenic contamination on various metrics of total and bioavailable arsenic concentration, soil nutrients, pH, and bacterial α-diversity (An indicator used in ecology to describe the species diversity within a single sample or local habitat. Alpha diversity measures the species richness and evenness within an individual community, reflecting the degree of species diversification in a specific area or sample). Duncan’s test was used to determine significant differences at *p* < 0.05. Bacterial α diversity indices were calculated in R v. 3.5.1 using the picante package [48]. We calculated the Shannon index as a proxy for bacterial alpha diversity [49]. Principal coordinates analysis (PCoA) in the vegan package was used to analyze dissimilarities between bacterial communities, and the adonis2 function was used to detect significant effects of time and degree of arsenic contamination on soil bacterial communities. Mantel.test was used to detect correlations between soil physical and chemical properties and bacterial communities. Pearson correlation was used to analyze the connectivity of microbial molecular ecological networks in relation to environmental factors.

## 3. Results

### 3.1. Total Arsenic and Bioavailable Arsenic Concentration

**Figure 1:**
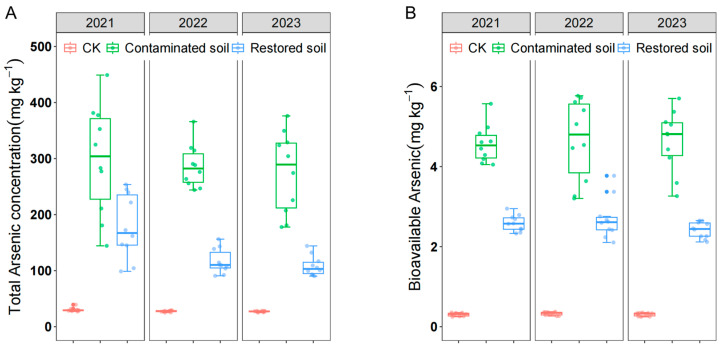
Total arsenic concentration (**A**) and bioavailable arsenic concentration (**B**) over time in arsenic−contaminated soil, *Pteris vittata* L. remediated soil, and control soil. Error bars represent standard deviation *(n* = 10).

Despite soil arsenic contamination in the study area persisting for over 20 years, the average arsenic concentration in contaminated soil remained close to 300 mg kg^−1^ (Figure 1A), far exceeding the national secondary standard limit of 40 mg kg^−1^. Both total and bioavailable arsenic concentrations showed little change from 2021 to 2023. Statistical analysis revealed significant differences in both total and bioavailable arsenic concentration among different treatments. Total arsenic concentration in arsenic-contaminated soil was significantly higher than in remediated and control soils, and arsenic concentration in *Pteris vittata* L. remediated soil was also significantly higher than in control soil (Figure 1A), indicating that *Pteris vittata* L. remediation can effectively remove arsenic from soil. Bioavailable arsenic showed a pattern of change that was almost identical to that of total arsenic (Figure 1B). Two-way ANOVA indicated that arsenic concentration had a significant effect on both total and bioavailable arsenic concentration. However, time only had a significant effect on total arsenic but not bioavailable arsenic concentration (Table 1).

### 3.2. Soil Nutrients

**Figure 2:**
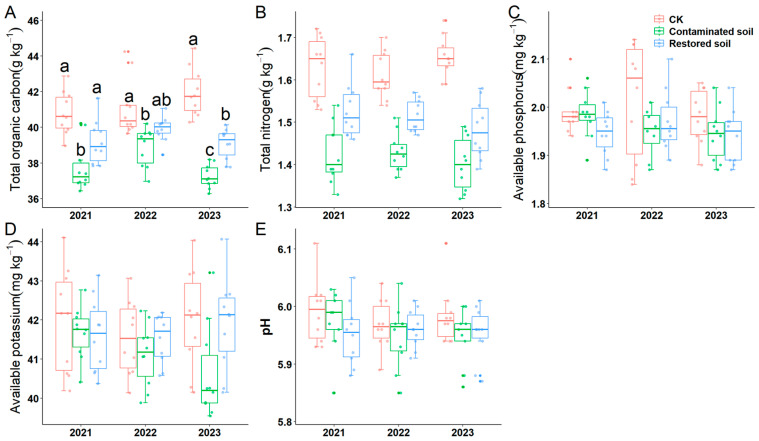
Response of soil physicochemical properties to arsenic-contamination and *Pteris vittata* L. remediation from 2021 to 2023 (**A**–**E**). (**A**) shows the changes in soil total organic carbon, (**B**) shows the changes in soil total nitrogen, (**C**) shows the changes in soil quick−acting phosphorus, (**D**) shows the changes in soil quick−acting potassium, and (**E**) shows the changes in soil pH. Different lowercase letters in A indicate significant differences (*p* < 0.05, Duncan’s test) among different treatments in each year. Error bars represent standard deviation (*n* = 10).

Two-way ANOVA indicated that arsenic contamination significantly affected soil total carbon, total nitrogen, available phosphorus, available potassium and pH. Moreover, time and the interaction between time and treatment were solely observed for soil total organic carbon (Table 1). Subsequent simple effects analysis revealed that soil total organic carbon in the *P. vittata* L. remediated soil treatments in 2021 and 2022 did not differ significantly from the control treatment. Soil total organic carbon in the *Pteris vittata* L. remediated soil treatment in 2022 also did not differ significantly from the arsenic-contaminated soil. Significant differences in total organic carbon content were observed across all other treatments (average value is 37.965 g kg^−1^, 39.388 g kg^−1^ and 41.278 g kg^−1^ in contaminated soil, restored soil and CK respectively, *F* = 62.656, *p* < 0.001) (Figure 2A). This indicates that arsenic contamination significantly reduces total soil organic carbon content, and *Pteris vittata* L. remediation can mitigate the negative impact of arsenic pollution on soil organic carbon. The variation pattern of soil total nitrogen content was generally similar to that of soil total organic carbon (average value is 1.419 g kg^−1^, 1.508 g kg^−1^ and 1.632 g kg^−1^ in contaminated soil, restored soil and CK respectively, *F* = 99.381, *p* < 0.001) (Figure 2B). Pairwise comparisons of the main effects demonstrated that total nitrogen content was significantly higher in the control soil than in the *Pteris vittata* L. remediated and arsenic-contaminated soils, which also differed significantly from each other (Supplementary Table S4). Similarly, pairwise comparison of the main effects revealed that the available phosphorus content in the control soil was significantly higher than that in the arsenic-contaminated soil, while no significant differences were observed among the other treatments (Figure 2C, Supplementary Table S4). The results for pH were consistent with those for available phosphorus (Figure 2E, Supplementary Table S4).

### 3.3. Soil Bacterial Diversity

**Figure 3:**
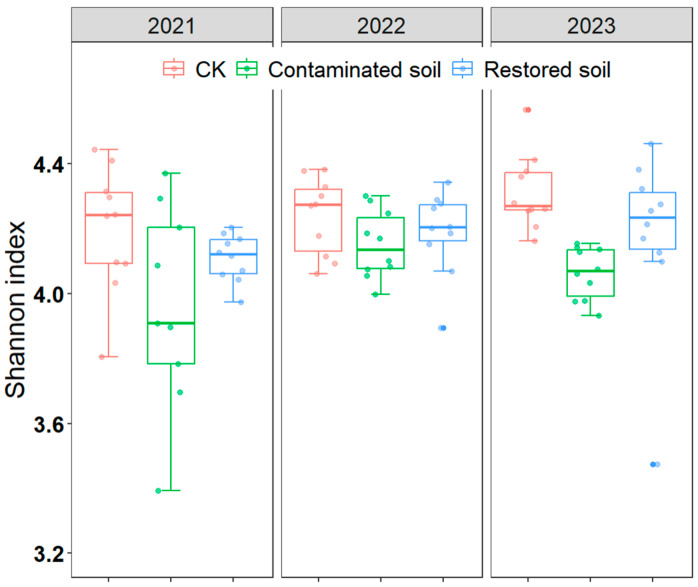
Changes in soil bacterial Shannon index over time at different treatments in arsenic−contaminated soil, *Pteris vittata* L. remediation soil and CK. Error bars represent standard deviation (*n* = 10).

BLAST (Version 2.14.1+) results indicated that Acidobacteriota, Bacteroidota, and Proteobacteria were the dominant bacterial phyla in the soil (each exhibiting relative abundances greater than 10% across the three treatments, Supplementary Figure S2). The relative abundance of Acidobacteriota, Nitrospirota, and Verrucomicrobiota was slightly higher in arsenic-contaminated soil than in the control soil, while Chloroflexi, Planctomycetota, and Proteobacteria displayed lower relative abundance (Supplementary Figure S2). Two-way ANOVA revealed that both arsenic contamination and time significantly affected the Shannon diversity index of bacteria, and there was no interaction between contamination and time (Table 1). Pairwise comparisons of the main effects indicated that the Shannon diversity index of the control treatment was significantly higher than that of the arsenic-contaminated soil treatment, and the Shannon diversity index for the *Pteris vittata* L. remediated soil treatment was also significantly higher than that of the arsenic-contaminated soil treatment, while the difference between the *Pteris vittata* L. remediated soil and control treatments was not significant (Figure 3, Supplementary Table S4).

### 3.4. Soil Bacterial Communities

**Figure 4:**
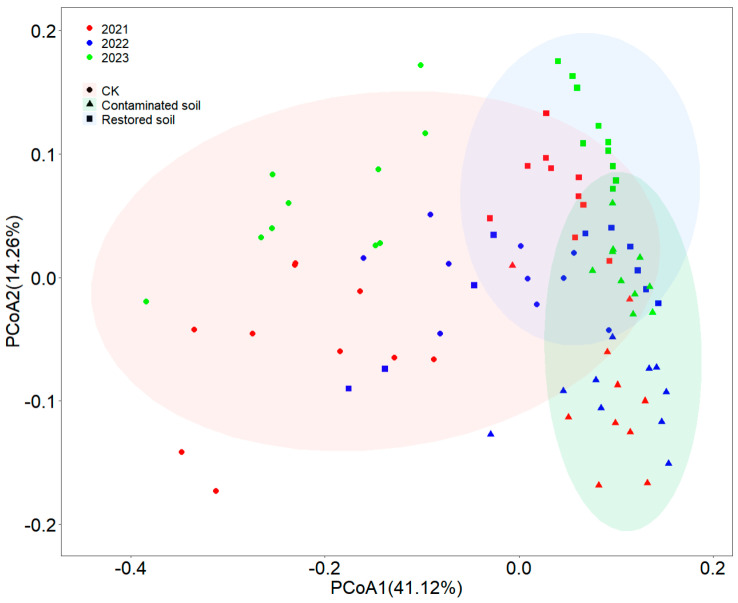
The Principal Coordinates Analysis (PCoA) results of bacterial communities are presented with symbols coded according to arsenic contamination and with different color contrasts representing different years. Specifically, red represents 2021, green represents 2022, and blue represents 2023. Different symbols denote different treatments, where circles represent control soil, triangles represent arsenic−contaminated soil, and squares represent soil restored with *Pteris vittata* L. Ellipses of different colors indicate the 95% confidence intervals of bacterial community distribution under different treatment conditions.

Principal coordinate analysis (PCoA) indicated that two components (PCoA1 and PCoA2) collectively explained 55.38% of the total variance (Figure 4). Although some overlap existed in bacterial communities among different treatments, clear differences were still observable among arsenic-contaminated, *Pteris vittata* L. remediated and control soils (Figure 4). Two-way PERMANOVA analysis demonstrated that both contamination and time significantly affected bacterial communities (*p* < 0.001), with significant interactive effects between contamination and time (*p* < 0.001) (Supplementary Table S5). Compared to arsenic-contaminated and *Pteris vittata* L. remediated soils, control soils showed relatively larger Bray–Curtis dissimilarity distances between samples and a more dispersed distribution of bacterial communities, while bacterial communities in arsenic-contaminated and *Pteris vittata* L. remediated soils exhibited a clustered distribution pattern (Figure 4). The Mantel test results showed that soil total carbon and total nitrogen were significant factors in shaping bacterial communities (Supplementary Table S6).

### 3.5. Soil Bacterial Molecular Ecological Networks

**Figure 5:**
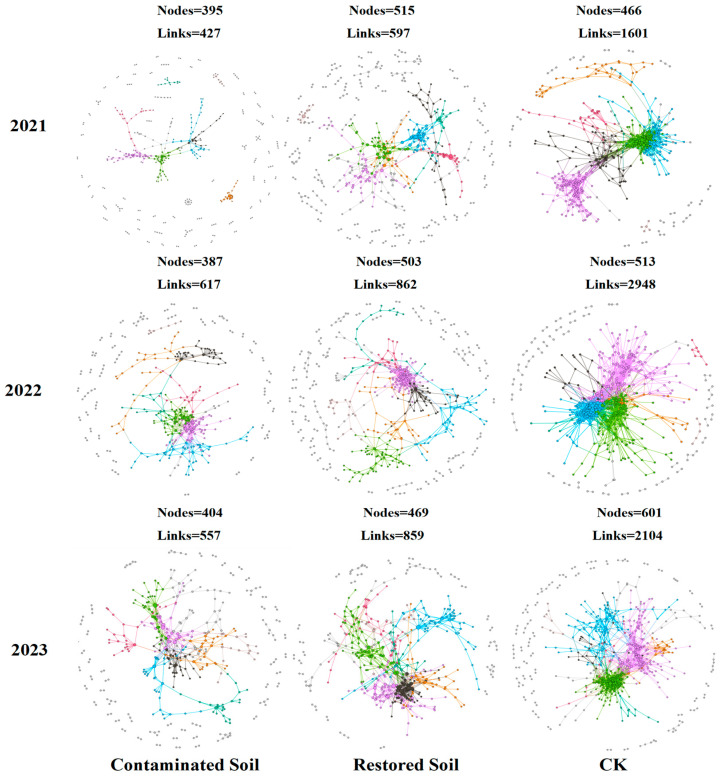
Bacterial molecular ecological networks succession in contaminated, remediated, and control soils over time. Visualization of constructed Molecular Ecological Networks in 3 years from 2021 to 2023. Large modules with ≥5 nodes are shown in different colours, and smaller modules are shown in gray.

Based on high-throughput sequencing data, we constructed nine soil bacterial community molecular ecological networks (Figure 5). The results presented in Table 2 showed that the average connectivity, average geodesic distance, average clustering coefficient, and modularity in the molecular ecological networks were all higher than the corresponding values in random networks, with R2 values greater than 0.86. This finding suggests that the networks constructed in this study exhibit scale-free, small-world, and modular network characteristics [46,50]. As increasing arsenic contamination levels, the bacterial community network exhibits a decreasing trend in the number of nodes, links, and average connectivity. The average number of nodes was 526, 495, and 395 in CK, *Pteris vittata* L. remediated soil, and arsenic-contaminated soil, respectively, while the average number of links was 2217, 772, and 533 in CK, *Pteris vittata* L. remediated soil, and arsenic-contaminated soil, respectively. Compared to arsenic-contaminated soil, *Pteris vittata* L. remediated soil exhibited an increase of approximately 25% and 91% in node and link quantity respectively. In contrast, the average geodesic distance and modularity showed an increasing trend with the degree of pollution (Figure 5 and Table 2). The average geodesic distance increased from 4.48 in the control soil to 6.84 in the arsenic-contaminated soil, and the modularity value increased from 0.54 in the control soil to 0.77 in the contaminated soil. As the pollution level increased, the connections between bacterial species became simpler, and the overall network structure of the soil bacterial community underwent significant changes (Figure 5).

**Figure 6:**
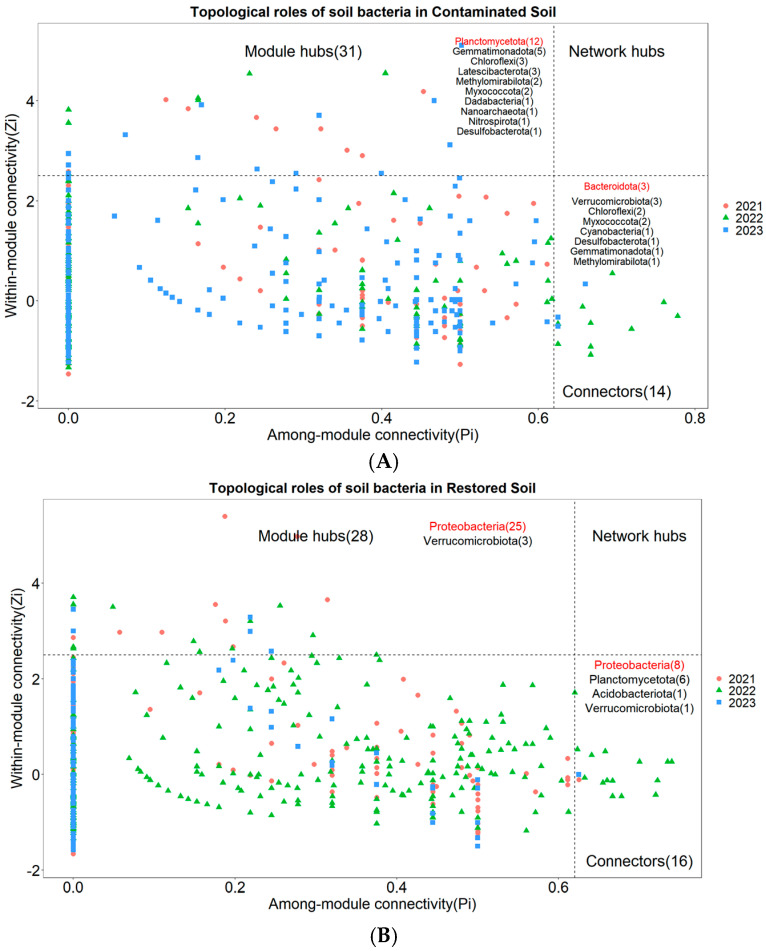

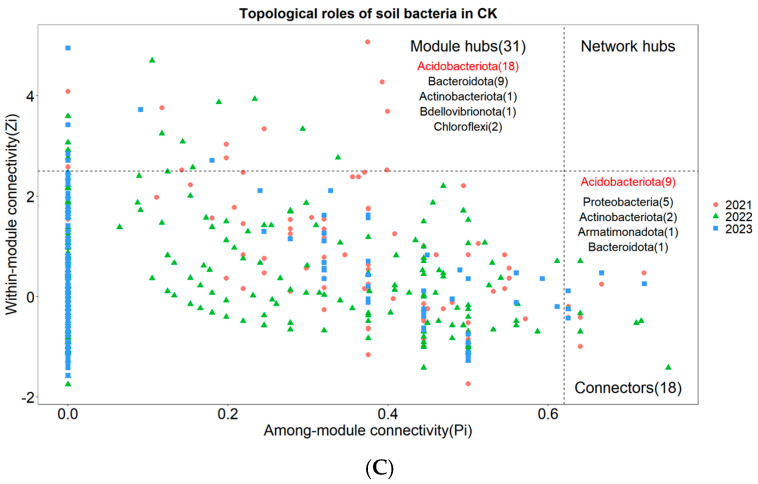
The Zi−Pi plot illustrates the distribution of Operational Taxonomic Units (OTUs) from different treatments ((**A**) for Arseni−contaminated Soil, (**B**) for Restored Soil, (**C**) for Control Soil) based on their topological roles. Various symbols represent the years 2021 to 2023, where circles denote 2021, triangles represent 2022, and squares represent 2023. The topological role of each OTU was determined based on the scatter plot of within−module connectivity (Zi) and among−module connectivity (Pi). The numbers in parentheses following Module hubs indicate the quantity of nodes falling within Module hubs, while the numbers following Connectors represent the quantity of nodes falling within Connectors. The bacterial phyla to which these nodes belong are also marked within their respective regions, with those labeled in red indicating dominant key species.

Nodes with Zi ≥ 2.5 or Pi ≥ 0.62 are generally considered to be key species that maintain the network structure [31]. None of the three treatments had network nodes in the Network hubs area (Figure 6A–C). In the arsenic-contaminated soil treatment, 3.62% of the nodes fell into the Connectors area and 8.01% of the nodes fell into the Module hubs area (Figure 6A). In the *Pteris vittata* L. remediated soil treatment, 3.18% of the nodes fell into the Connectors area and 5.57% of the nodes fell into the Module hubs area (Figure 6B). In the control treatment, 3.84% of the nodes fell into the Connectors area and 6.61% of the nodes fell into the Module hubs area (Figure 6C). The key species across the three treatments were notably different. The key species with the highest connectivity in arsenic-contaminated soil was Planctomycetota, the key species with the highest connectivity in *Pteris vittata* L. remediated soil was Proteobacteria, and the key species with the highest connectivity in the control treatment was Acidobacteriota (Figure 6A–C).

To analyze the relationship between microbial molecular ecological networks and soil environmental factors, we performed a Pearson correlation analysis between connectivity and soil physicochemical properties. The results indicated that soil total organic carbon and total nitrogen contents were significantly correlated with network connectivity (Supplementary Figure S3). This indicates that soil carbon and nitrogen play an important role in determining microbial network interactions.

## 4. Discussion

### 4.1. Arsenic Concentrations and Soil Nutrients

During the 3-year observation period of this study, total arsenic concentrations did not exhibit a significant decreasing trend in any of the treatments, indicating that arsenic in soils remains stable due to its exceptionally slow rate of mobility and transformation. More than ten years ago, Xiong et al. [37] detected arsenic concentrations of around 300–350 mg kg^−1^ in bulk soils in this region. However, over a decade later, our results showed that the average total arsenic concentrations in the arsenic-contaminated soils still exceeded 300 mg kg^−1^ on average, with barely any noticeable decrease. Relying on natural attenuation to remediate arsenic contamination is unlikely achievable in a short period, as previous studies have reported that arsenic can persist in soils for thousands of years [51,52]. Therefore, anthropogenic intervention is almost the only viable approach for remediating arsenic-contaminated soils. Among these, phytoremediation is considered one of the most effective options. Bioavailable arsenic refers to the arsenic that can be absorbed and utilized by living organisms. Therefore, compared with total arsenic, bioavailable arsenic is more likely to be the primary factor contributing to the decline in bacterial diversity and simplification of network structure in this study. Studies have shown that bioavailable arsenic is significantly negatively correlated with microbial biomass [53].

Continuous monitoring of the long-term impacts of field arsenic contamination on soil nutrients is important for assessing changes in soil quality and ecosystem functions. Our results showed that long-term arsenic contamination negatively affected most soil nutrients examined. This aligns with findings from several previous studies. For example, Xiong et al. [37] found that soils from areas with high arsenic contamination had substantially lower organic matter, total nitrogen and phosphorus contents compared to control soils. Li et al. [13] also observed significantly lower total organic carbon in severely contaminated soils than mildly contaminated ones. Such negative effects on soil nutrients can be reasonably explained by the inhibitory effects of long-term arsenic contamination on soil microorganisms [53], including functional microbial groups or genes [54,55,56]. For instance, Subrahmanyam et al. [54] found in a short-term laboratory experiment that copy numbers of the amoA gene and potential nitrification rates significantly declined with increasing arsenic levels. Arsenic contamination also greatly reduces microbial biomass and enzyme activities [57,58]. Given the crucial roles of soil microbes in nutrient cycling [59], decreases in microbial abundance and enzyme activities inevitably lead to lower turnover rates of soil nutrients. Therefore, it is understandable that we observed significantly lower levels of total carbon, total nitrogen and available phosphorus in the arsenic-contaminated soils. The soil nutrients to some extent reflect the health and quality of the soil. In this study, most nutrient levels in the *Pteris vittata* L. remediation plots were in an intermediate state, higher than the arsenic-contaminated soil but lower than the control soil. This highlights the important role of *Pteris vittata* L. remediation in improving soil quality. The underlying mechanism may be related to the substantial amount of *Pteris vittata* L. residues remaining in the remediation plots and its modulation of the rhizosphere microbial communities. Recent literature emphasizes the multifaceted role of *Pteris vittata* L. in enhancing soil quality through its interactions with rhizosphere microbes. By accumulating pollutants such as arsenic and cultivating beneficial microbial populations [18,20], modification of soil chemistry via root exudates, and enhancing microbial diversity through symbiotic relationships with fungi [60], *Pteris vittata* L. becomes an important mediator of phytoremediation, enhancing soil nutrients and ecosystem restoration capability in contaminated environments. These findings underscore the importance of incorporating plant-microbe interactions into effective soil remediation and management strategies.

Arsenic contamination also markedly decreased soil pH, likely due to the higher proportions of Nitrospirota (2.18%, 1.72% and 0.90% in arsenic-contaminated soil, *Pteris vittata* L. remediated soil and CK, respectively) and Acidobacteriota (32.49%, 33.06% and 30.83% in arsenic-contaminated soil, *Pteris vittata* L. remediated soil and CK, respectively) in the arsenic-contaminated soils compared to the control (Supplementary Figure S1). This is attributed to the ability of Nitrospirota to promote nitrification and produce more NO_3_^−^, while Acidobacteriota can enhance soil acidity by oxidizing sulfur minerals [12].

In summary, one of the objectives of this study was to assess whether arsenic accumulation and its bioavailability are linked with changes in soil nutrient status. The concomitant increase in total and bioavailable As with declines in TOC, TN and AP indicates that long-term arsenic stress reduces soil fertility and nutrient cycling capacity, which are fundamental components of soil health. This finding addresses our research goal by showing how As contamination directly compromises soil quality. Notably, plots remediated with *Pteris vittata* L. exhibited relatively higher nutrient levels compared to contaminated plots, suggesting that phytoremediation partially alleviated the negative effects of arsenic on soil fertility and contributed to the gradual recovery of soil health.

### 4.2. Bacterial Diversity and Community Composition

Shannon diversity indices are commonly used to evaluate biodiversity. In this study, the control soils had significantly higher Shannon indices than the other two treatments, indicating that arsenic contamination substantially reduced bacterial diversity. Compared to the arsenic-contaminated soils, the *Pteris vittata* L. remediated soils showed significantly higher Shannon indices, suggesting that *Pteris vittata* L. remediation over several years facilitated the recovery of bacterial diversity. The conclusion that arsenic contamination decreases soil microbial diversity has been supported by many previous studies [20,28,61]. This is primarily attributed to the high sensitivity and low tolerance of most soil microbes to arsenic contamination, even at relatively low exposure levels [57,62]. Under extreme arsenic pollution, as in this study (arsenic concentration up to 300 mg kg^−1^), many microbial species were eliminated due to their inability to adapt, resulting in reduced microbial diversity. Given the close links between soil microbial diversity and ecosystem services [63,64,65], the loss of diversity would inevitably impair soil ecosystem functions.

Under prolonged arsenic stress, some sensitive bacterial species were inhibited or even eliminated, while certain arsenic-resistant species persisted and proliferated, thereby altering the original bacterial communities and forming new community structures. In this study, the arsenic-contaminated, *Pteris vittata* L. remediated and control soils showed minimal differences in bacterial phyla compositions (Supplementary Figure S1). However, their bacterial communities still exhibited significant distinctions (Figure 4), indicating greater differences at lower bacterial taxonomic levels. It is widely recognized that arsenic contamination can markedly influence soil microbial communities [20]. Here, we found that the bacterial communities in the arsenic-contaminated soils were more closely clustered with smaller dissimilarity distances compared to the control. Community dissimilarity is often used to describe beta diversity, with larger dissimilarity distances indicating higher beta diversity. Our results suggest that arsenic contamination may reduce the beta diversity of bacterial communities (Figure 4). Lower beta diversity leads to the homogenization of species compositions across habitats, rendering the habitats more vulnerable and more prone to ecosystem collapse [66].

Overall, another aim of this study was to investigate how microbial diversity and community structure respond to arsenic contamination and phytoremediation. The reduction in bacterial α-diversity and homogenization of community composition under arsenic stress indicate a loss of functional redundancy and adaptability, both critical for soil health. However, the remediated soils displayed increased Shannon diversity and a community structure more similar to the control, highlighting that phytoremediation mitigates the deterioration of microbial diversity and supports the restoration of soil health potential.

### 4.3. Bacterial Molecular Ecological Networks

Molecular ecological networks are structural models that depict potential species interactions at the community level, constructed from occurrence and abundance data. They reflect the functional and structural characteristics of communities in ecosystems and represent a powerful and promising tool for biomonitoring and assessment [67,68]. In molecular ecological networks, nodes denote species in the community, and links represent associations between them [67]. In this study, the numbers of nodes, links and average connectivity were lowest in the contaminated soils, moderate in the restored soils and highest in the control soils over the three years. This indicates that arsenic contamination reduced bacterial interactions and network complexity, and altered the overall network structure. The simplification of bacterial networks under arsenic stress may lower network stability [69], weakening the resistance of the whole soil ecosystem against environmental changes and threatening soil health [31,70]. Compared to the contaminated soils, *Pteris vittata* L. restoration of contaminated soils substantially increased bacterial node numbers (by approximately 25%) and links (by approximately 91%). The increase in node numbers aligns with the elevated bacterial diversity. The sharp rise in links suggests that *Pteris vittata* L. restoration could markedly strengthen connections between bacterial species and enhance network complexity. The average path distance and modularity values increased with arsenic contamination levels, indicating that arsenic contamination increased distances between nodes and modularization. These results are highly similar to those of Li et al. [71], who found average path distances and modularity values increased with heavy metal contamination intensities.

Nodes classified as module hubs, network hubs, and connectors play important roles in connecting modules and within their own modules. As key species, they influence the connectivity and stability of bacterial network structures [50]. Our results showed that the key species were markedly different across treatments, suggesting that arsenic contamination tremendously altered the core bacterial species maintaining network structures. The changes in core species may be attributed to variations in arsenic concentration levels and environmental conditions across various treatments. Various microorganisms exhibit differing levels of tolerance and adaptability to arsenic concentrations, which can lead to different microbial species becoming dominant under different levels of arsenic pollution and other environmental conditions. For instance, some arsenic-resistant microorganisms possess the ars operon. This operon typically includes genes such as arsR (a repressor), arsB (an efflux permease), and arsC (an arsenate reductase). These genes facilitate detoxification by expelling arsenite or reducing arsenate to less harmful forms [72]. Some bacteria can also utilize methylation pathways to convert arsenic into less toxic methylated forms, further reducing its bioavailability in the environment [73]. These arsenic-tolerant microorganisms gradually become the core species that maintain the network structure. The study by Jiao et al. [74] also indicated that core microbial assemblages play vital ecological roles in maintaining complex links between bacterial groups. Further investigations are needed to understand how such shifts in key species could impact soil ecosystems, but it is foreseeable that changes in core species would likely affect the ecological processes of soil carbon, nitrogen and nutrient cycling [11,74,75]. Moreover, the core microbiota in heavily arsenic-contaminated soil may be associated with the decomposition, activation, or immobilization of arsenic [76]. Isolating and culturing these microorganisms and reintroducing them into arsenic-contaminated environments could potentially offer an avenue for addressing arsenic pollution issues.

Our Pearson correlation analyses revealed that soil total organic carbon and total nitrogen were key determinants of bacterial molecular ecological networks. Organic carbon is the main source of energy for microorganisms, and the amount of carbon in the soil directly affects the growth and reproduction of microorganisms. Total nitrogen provides the nitrogen necessary for microorganisms to synthesize proteins and nucleic acids. Different types of microorganisms have varying needs for carbon and nitrogen, and their competitive and cooperative relationships affect the overall microbial network structure [77]. Several recent studies have emphasized the complex interactions between soil environmental factors and microbial molecular ecological networks. The total organic carbon/total nitrogen (TOC/TN) ratio has been identified as a key driver of microbial community assembly processes. It shapes the diversity and complexity of bacterial networks, with different ratios promoting distinct community structures. For instance, a balanced TOC/TN ratio can enhance the stability and connectivity of microbial networks, facilitating cooperative interactions among bacteria [78,79]. Wu et al. [80] conducted a study in a forested wetland ecotone in China and found that key fungal network modules were notably influenced by total nitrogen and water-soluble potassium levels, while bacterial modules were positively driven by total nitrogen, magnesium, and sodium. These studies highlight the importance of soil nutrients in microbial network dynamics. Understanding these relationships is essential for effectively managing soil health and ecosystem services. These findings underscore the need for continued exploration into how specific environmental conditions influence microbial interactions within various ecosystems.

We further aimed to explore whether arsenic contamination alters microbial interaction networks, as such structural changes are closely tied to ecosystem stability. The simplified microbial networks in contaminated soils—fewer nodes and links with lower connectivity—reflect weakened microbial interactions and reduced resilience, indicative of compromised soil health. In contrast, the remediated soils exhibited denser and more complex networks, with increased nodes, links, and keystone taxa, pointing to enhanced interaction stability and resilience. These results demonstrate that Pteris vittata phytoremediation not only mitigates network simplification but also fosters conditions conducive to the recovery of soil health.

### 4.4. Practical Implications, Limitations, and Future Research

Our field results indicate that *Pteris vittata* L. phytoremediation can partially ameliorate arsenic-induced declines in soil nutrient status and can promote increases in microbial diversity and network complexity. We therefore recommend a staged implementation path from targeted pilots to scaled deployment, accompanied by an integrated monitoring program that tracks total and bioavailable As, core physico-chemical properties (TOC, TN, AP, pH), and biological indicators (microbial diversity, network metrics, and selected functional assays). Pilot trials should test complementary amendments and harvesting regimes, and harvested biomass must be managed as contaminated waste in compliance with local regulations. Such an adaptive, evidence-based approach will help optimize remediation efficacy and verify the robustness of soil-health recovery under real environmental variability.

While our findings demonstrate that *Pteris vittata* L. phytoremediation can mitigate arsenic-induced soil degradation and partially restore soil health, several limitations of this study should be acknowledged. First, the field experiment was conducted in a specific subtropical region with loamy clay Cambisols, and the results may not be directly generalizable to other soil types or climatic conditions. Second, our analyses focused primarily on taxonomic composition and ecological network patterns; without metagenomic or functional gene profiling, the underlying microbial mechanisms driving remediation remain insufficiently resolved. Third, functional soil health indicators such as enzyme activities and microbial biomass related to nutrient cycling were not evaluated, which constrains our ability to link community changes directly to ecosystem processes. Another limitation is that our microbial co-occurrence networks were inferred using Pearson correlations of relative abundance data. Such correlation-based approaches can be influenced by the compositional nature of sequencing data, which may introduce spurious associations. As a result, the networks presented here should be interpreted as indicative of potential co-occurrence patterns rather than conclusive ecological interactions. Future studies should consider applying compositionally aware methods (e.g., SPIEC-EASI or SparCC) to strengthen the robustness of network inference. Future research should therefore include long-term and multi-site studies across different soils and climates, incorporate functional assays (e.g., enzyme activities, microbial biomass, key functional genes), and integrate metagenomic or metatranscriptomic approaches to elucidate microbial mechanisms. Such efforts will provide a more comprehensive and mechanistic understanding of how phytoremediation contributes to sustainable soil health recovery.

## 5. Conclusions

This three-year field study demonstrated that phytoremediation with *Pteris vittata* L. can effectively restore bacterial diversity and network complexity in long-term arsenic-contaminated soils. Compared to untreated contaminated soils, *Pteris vittata* L. remediated soils showed recovery of soil nutrients, increased bacterial diversity, more complex bacterial ecological networks, and shifts in bacterial community composition toward the control soils. The enhancement of biodiversity and network complexity signals ecological restoration and improved soil health. This highlights the positive effects of *Pteris vittata* L. restoration on the improvement of long-term arsenic-contaminated soil ecosystems. However, total arsenic concentrations remained high and unchanged in both contaminated and remediated soils over the 3-year period, indicating very slow natural attenuation of arsenic. Further research is needed to promote arsenic removal while preserving ecological functions. Isolating core microbial taxa from severely arsenic-contaminated areas and reintroducing them into arsenic-contaminated environments may offer a fresh perspective on addressing arsenic pollution.

## Figures and Tables

**Table 1:** Two-way ANOVA to analyze the effects of arsenic contamination and time on soil total arsenic and bioavailable arsenic content, soil physicochemical properties, and bacterial diversity.

	Total Arsenic Concentration (mg kg^−1^)	Bioavailable Arsenic Concentration (mg kg^−1^)	Total Organic Carbon (g kg^−1^)	Total Nitrogen (g kg^−1^)	Available Phosphorus (mg kg^−1^)	Available Potassium (mg kg^−1^)	Soil pH	Shannon Index
Contaminate	*F* = 223.497 ***	*F* = 575.883 ***	*F* = 62.656 ***	*F* = 99.381 ***	*F* = 4.448 *	*F =* 3.92 *	*F* = 3.453 *	*F* = 8.691 ***
Time	*F* = 3.848 *	ns	*F* = 3.195 *	ns	ns	ns	ns	*F* = 4.204 *
Contaminate × Time	ns	ns	*F* = 3.287 *	ns	ns	ns	ns	ns

Note: Symbols * indicate significance at *p* < 0.05, *** indicate significance at *p* < 0.001, statistical difference among treatment groups according to Duncan’s test; ns indicates no significant difference.

**Table 2:** Comparison of topological properties of molecular ecological and random networks in arsenic-contaminated, remediated, and control soil bacteria from 2021–2023.

		Molecular Ecological Network									Random Network		
Time	Treatments	Gene Numbers	Similarity Threshold	R^2^	Nodes	Links	Average Connectivity	Average Geodesic Distance	Average Clustering Coefficient	Modularity	Average Geodesic Distance	Average Clustering Coefficient	Modularity
2021	Contaminated soil	799	0.890	0.936	395	427	2.162	7.446	0.084	0.877	6.292 ± 0.213	0.004 ± 0.003	0.784 ± 0.009
	Restored Soil	927	0.880	0.920	515	597	2.318	7.186	0.105	0.857	6.192 ± 0.142	0.004 ± 0.002	0.757 ± 0.007
	CK	860	0.910	0.909	466	1601	6.871	5.183	0.153	0.587	3.245 ± 0.027	0.065 ± 0.006	0.327 ± 0.005
2022	Contaminated soil	936	0.910	0.884	387	617	3.189	6.525	0.092	0.683	4.296 ± 0.068	0.019 ± 0.005	0.582 ± 0.007
	Restored Soil	901	0.890	0.918	503	862	3.427	6.115	0.144	0.706	4.085 ± 0.065	0.031 ± 0.006	0.552 ± 0.006
	CK	999	0.930	0.865	513	2948	11.493	3.690	0.219	0.470	2.915 ± 0.021	0.106 ± 0.005	0.221 ± 0.003
2023	Contaminated soil	944	0.910	0.937	404	557	2.757	6.555	0.144	0.750	4.763 ± 0.084	0.011 ± 0.004	0.652 ± 0.007
	Restored Soil	867	0.910	0.869	469	859	3.663	5.907	0.107	0.716	4.229 ± 0.051	0.018 ± 0.005	0.535 ± 0.006
	CK	960	0.910	0.883	601	2104	7.002	4.576	0.092	0.562	3.367 ± 0.026	0.047 ± 0.004	0.328 ± 0.004

## Data Availability

The bacterial sequences recovered in this study were deposited in the NCBI GenBank Sequence Read Archive (SRA) under accession numbers SRR28131172.

## Supplementary Materials

The following supporting information can be downloaded at: https://www.mdpi.com/article/10.3390/microorganisms13102316/s1, Figure S1 Study site in this study in Dengjia Tang Town, Chenzhou City, Hunan Province, South China. Figure S2: Comparison of the classification of dominant bacterial phyla among treatments; Figure S3: Pearson’s correlation analysis of connectivity of microbial molecular ecological networks with soil nutrients and pH values. Figure S3A Soil total organic carbon, Figure S3B Soil total nitrogen, Figure S3C Soil quick-acting phosphorus, Figure S3D Soil quick-acting potassium, and Figure S3E Soil pH. Table S1: Criteria for determining soil texture; Table S2: Soil texture among treatments (n = 4); Table S3: Vegetation information in the three treatments; Table S4: Results of pairwise comparisons among treatments after the main effect was significant; Table S5: Two-way PERMANOVA to analyze the effects of arsenic contamination and time on soil bacterial community. Symbols * indicate significance at *p* < 0.05, ** indicate significance at *p* < 0.01, *** indicate significance at *p* < 0.001; Table S6: The environmental factors that correlated with bacterial communities are listed below. The correlations (r2) and significance (P) were determined by mantel.test between the bacterial communities and environmental variables.
