# Supplementary material for: The Remediation of Arsenic-Contaminated Soil by Pteris vittata L. Facilitates the Recovery of Soil Bacterial Diversity and Network Complexity
Source: Microorganisms. 2025 Oct 7;13(10):2316. doi: 10.3390/microorganisms13102316 (PMC12566545; doi:10.3390/microorganisms13102316)
Supplement: Supplementary file 1 [file microorganisms-13-02316-s001.zip › microorganisms-3895870-supplementary.pdf]

SUPPORTING INFORMATION

The remediation of arsenic-contaminated soil by *Pteris vittata L.* facilitates the recovery of soil bacterial diversity and network complexity

Feng Li[1,2], Jinhua Liu[1,2], Tao Tian[1,2], Bin Deng[1,2*], Haifeng Xiao[1,2,*]

[1]*School of Chemistry and Environmental Science, Xiangnan University, Chenzhou 423000, China*

[2]*Hunan Provincial Key Laboratory of Xiangnan Rare-Precious Metal Compounds and Application s, Chenzhou, 423000, China*

** Corresponding author. Email: hfxiao@xnu.edu.cn, Tel: +07352653035; dengbinxnu@163.com, Tel.:+07352865996*

This study used the hydrometer method to determine soil particle composition.

Soil Particle Determination Steps:

1. First, weigh 10 g of air-dried soil that has passed through a 2 mm sieve and place it in a beaker. Dry it until a constant weight is achieved to calculate the soil moisture conversion coefficient.
2. Weigh 50 g of air-dried soil that has passed through a 2 mm sieve and place it in a 250 ml beaker.
3. Soil Dispersion: Based on the soil's pH value, add sodium hydroxide solution to the beaker and add water to bring the volume of the suspension to 230 ml. Stir thoroughly with a glass rod and heat on a hot plate to boil for 1 hour, stirring continuously to prevent soil particles from settling into hard lumps at the bottom of the beaker.
4. Suspended Liquid Filtration: Place a funnel on a 1000 ml graduated cylinder and insert a 0.25 mm aperture wash sieve into the funnel. After the suspension cools, stir the suspension in the beaker thoroughly and wash it into the sieve with water. Gently wash and rinse the soil particles in the sieve with a rubber-tipped glass rod and water until the filtered water is no longer cloudy. Also, take care not to exceed 1000 ml in the graduated cylinder. Place the graduated cylinder containing the suspension on a stable workbench to avoid vibrations. Wash the sand particles remaining in the sieve into a pre-weighed beaker with water and dry at 105°C until a constant weight is achieved.
5. Temperature Measurement: Suspend a thermometer in a graduated cylinder containing 1000 ml of water, and place it next to the graduated cylinder containing the suspension to record the water temperature, which represents the temperature of the suspension.
6. Determination of Suspension Density: Stir the suspension for 1 minute, ensuring that the porous disk does not rise above the liquid surface to avoid foaming (suspensions with high organic matter content may foam during stirring, affecting hydrometer readings; therefore, a few drops of ethanol can be added to the suspension before placing the hydrometer). After stirring, let it sit for 1 minute and then place the soil hydrometer into the suspension. Record the reading where the hydrometer

aligns with the curved liquid surface. Measure the content of particles <0.05 mm, let it sit for 5 minutes, then measure the content of particles <0.02 mm. After 8 hours, measure the content of particles <0.002 mm. Refer to the temperature correction table for the soil hydrometer and calculate the corrected reading, which represents the cumulative content of particles smaller than the measured size.

Result Calculation Methods:

1. Soil moisture conversion coefficient calculation formula: K=m/m1, where K is the moisture conversion coefficient; m is the mass of dried soil; m1 is the mass of air-dried soil.
2. Calculation formula for each particle size content:

For the 2.0 mm - 0.25 mm particle size content (%) = m2/m×100;

For the content of particles below 0.05 mm, less than a certain particle size content (%) = m3/m×100;

where m2 is the mass of dried soil for the 2.0 mm - 0.25 mm size, m3 is the hydrometer reading corrected for particles below a certain size, and m is the mass of dried soil.

3. Dispersion agent mass correction formula:

A=(C×V×0.04/m)×100, where A is the percentage of the dispersion agent in the dried soil mass, %; C is the concentration of the dispersion agent solution, mol L^{-1}; V is the volume of the dispersion agent solution; m is the mass of dried soil, g, and 0.04 is the molar mass of sodium hydroxide, g $mmol^{-1}$.

4. Calculation formulas for each particle size content (%):

①Clay (<0.002 mm) content (%) = <0.002 mm particle size content (%) - A%;

②Silt (0.02 mm - 0.002 mm) content (%) = <0.002 mm particle size content (%) - <0.002 mm particle size content (%);

③Silt (0.05 mm - 0.02 mm) particle size content (%) = <0.05 mm particle size content (%) - <0.02 mm particle size content (%);

④Fine sand + very fine sand (0.25 mm - 0.05 mm) particle size content (%) = 100 - [2.0 mm - 0.25 mm particle size content (%) + 0.05 mm - 0.02 mm particle size content (%) + 0.02 mm - 0.002 mm particle size content (%) + <0.002 mm particle size content (%)];

⑤Sand (2.0 mm - 0.05 mm) particle size content (%) = 2.0 mm - 0.25 mm particle size content (%) + 0.25 mm - 0.05 mm particle size content (%);

⑥Sand (2.0 mm - 0.02 mm) content (%) = 2.0 mm - 0.05 mm particle size content (%) + 0.05 mm - 0.02 mm particle size content (%).

Table S1

Criteria for determining soil texture

Category	Name	Clay（<0.002mm）	Silt（0.02-0.002mm）	Sand（2.0-0.02mm）
Sandy soil	Sandy soil	0-15	0-15	85-100
	Sandy loam	0-15	0-45	55-85
Loamy soil	Loam	0-15	30-45	40-55
	Silty loam	0-15	45-100	0-55
Clay loam	Sandy clay loam	15-25	0-30	55-85
	Clay loam	15-25	20-45	30-55
	Silty clay loam 土	15-25	45-85	0-40
Clay	Sandy clay	25-45	0-20	55-75
	Loamy clay	25-45	0-45	10-55
	Silty clay	25-45	45-75	0-30
	Clay	45-65	0-35	0-55
	Heavy clay	65-100	0-35	0-35

Table S2

Soil texture among treatments (n=4).

treatments	Sand %<br>（2-0.02mm）	Silt %<br>（0.02-0.002）	Clay %<br>（<0.002mm）	Water content %
CK	32.21±0.34	42.98±0.26	24.81±0.09	38.67±0.89
Restored Soil	31.88±0.18	43.04±0.32	25.08±0.12	35.56±1.02
Contaminated Soil	31.65±0.23	43.37±0.37	24.98±0.21	35.19±1.12

According to the measured data corresponding to the above table (Table S1) query found that the soil texture in our sample plots belongs to loamy clay.

Table S3

Vegetation information in the three treatments.

Plant family	Plant species	CK	Restored Soil	Contaminated Soil
Pteridaceae	*Pteris cretica*	+	+	+
	Pteris vittata L.	-	+*	-
Blechnaceae	*Woodwardia japonica*	+	+	+
The lypteridaceae	*Cyclosorus arasiticus*	+	+	+
Moraceae	*Fatoue villose*	+	+	+
Urticaceae	*Boehmeria nivea*	+*	+	+*
Amaranthaceae	*Altemanthera philoxeroides*	+*	+*	+*
Polygonaceae	*Polygonum sp.*	+	+	+
Cruciferae	*Brassica sp.*	+	+	+
Myrtaceae	*Eucalyptus sp.*	+	+	+
Solanaceae	*Physalis alkekehgi*	+	+	-
	Solanum nigrum	+	+	+
Convolvulaceae	*Ipanoea batatas*	+	+	+
Plantaginaceae	*Plantago major*	+	+	+
Compositae	*Youngia japonica*	+*	+	+
	Eclipta prostrata	+	+	+
	Turczaninowia fastigiata	+*	+	+
	Bidens pilosa	+	+	-
	Conyza Canadensis	+	+	+
	Erigeron annuus	+	+	+
Gramineae	*Echinochloa crusgalli*	+	+	+
	Eleusine indica	+*	+*	+*
	Paspalum sp.	+	+*	+*
	Miscanthus floridulu	+	-	-
	Zeamays	+	+	+
Crassulaceae	*Sedum bulbiferum*	+	+	+

* indicate dominant plant species in the field plots. + means present, - indicate absent.

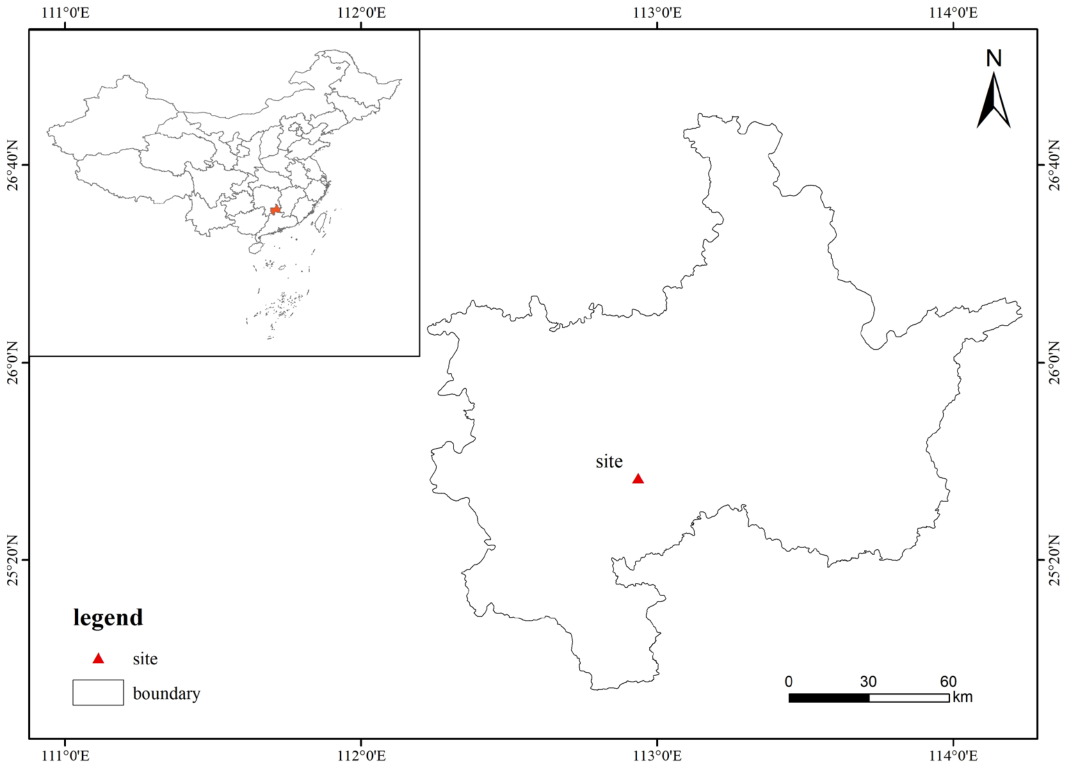

Fig. S1 Study site in this study in Dengjia Tang Town, Chenzhou City, Hunan Province, South China.

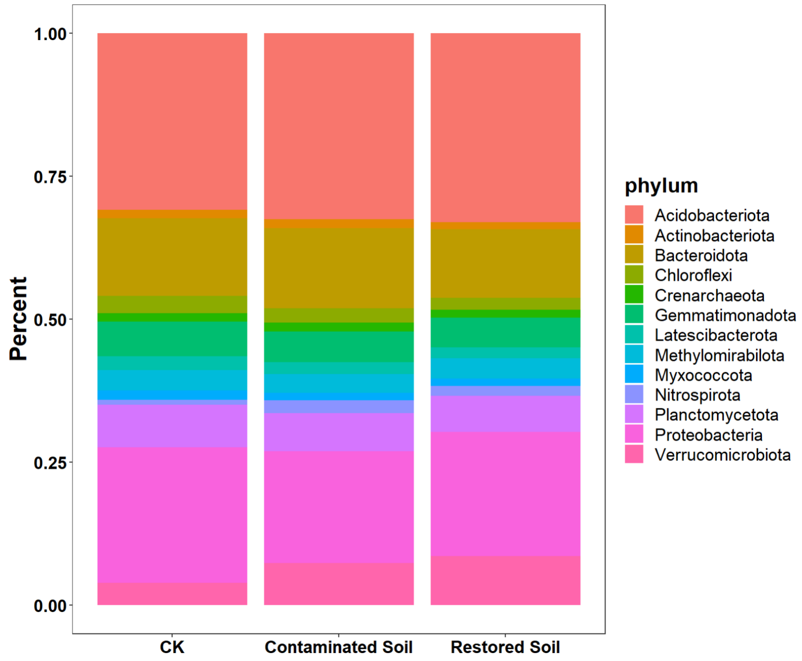

Fig. S2 Comparison of the classification of dominant bacterial phyla among treatments.

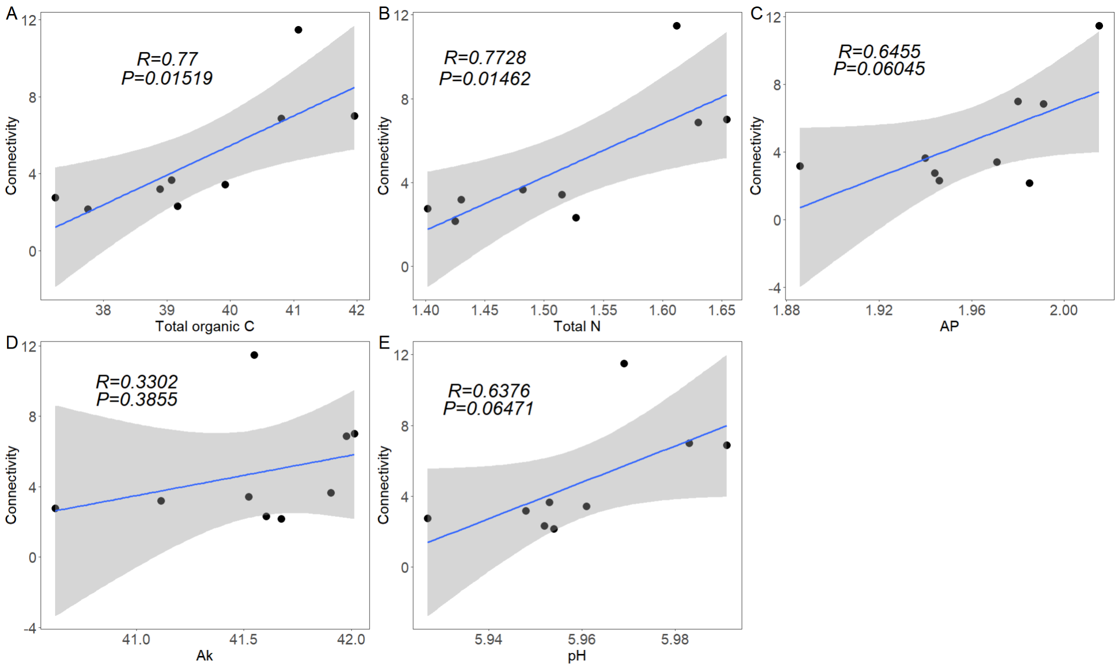

Fig. S3 Pearson's correlation analysis of connectivity of microbial molecular ecological networks with soil nutrients and pH values. Fig. S2A Soil total organic carbon, Fig. S2B Soil total nitrogen, Fig. S2C Soil quick-acting phosphorus, Fig. S2D Soil quick-acting potassium, and Fig. S2E Soil pH.

Table S4

Results of pairwise comparisons among treatments after the main effect was significant.

	Arsenic concentration (mg kg^{-1})	Bioavailable Arsenic concentration (mg kg^{-1})	Total organic carbon (g kg^{-1})	Total nitrogen (g kg^{-1})	Available phosphorus (mg kg^{-1})	Available potassium (mg kg^{-1})	Soil pH	Shannon index
Contaminated soil-CK	**P < 0.001**	**P < 0.001**	**P < 0.001**	**P < 0.001**	**P = 0.045**	P = 0.094	**P = 0.048**	**P < 0.001**
Restored soil-CK	**P < 0.001**	**P < 0.001**	**P < 0.001**	**P < 0.001**	P = 0.180	P = 1.000	P = 0.281	P = 0.324
Restored soil-Contaminated soil	**P < 0.001**	**P < 0.001**	**P < 0.001**	**P < 0.001**	P = 1.000	P = 0.285	P = 1.000	**P = 0.037**

Table S5

Two-way PERMANOVA to analyze the effects of arsenic contamination and time on soil bacterial community. Symbols * indicate significance at $P < 0.05$, ** indicate significance at $P < 0.01$, *** indicate significance at $P < 0.001$.

	Bacterial community
Contaminate	F=19.5878***
Time	F=10.3423***
Contaminate×Time	F=2.4926**

Table S6

The environmental factors that correlated with bacterial communities are listed below. The correlations (r^2) and significance (*P*) were determined by mantel.test between the bacterial communities and environmental variables.

Environmental variables	r^2	*P*
Total carbon	0.1671	0.008
Total nitrogen	0.2869	0.001
Available phosphorus	0.0007	0.422
Available potassium	0.0111	0.398
pH	0.0317	0.281
